# Asciminib monotherapy in patients with *BCR::ABL1* T315I-mutated chronic-phase chronic myeloid leukemia: phase 1 trial final results

**DOI:** 10.1038/s41375-026-02972-9

**Published:** 2026-07-16

**Authors:** Jorge E. Cortes, Delphine Rea, Michael J. Mauro, Andreas Hochhaus, Dong-Wook Kim, Koji Sasaki, Fabian Lang, Michael C. Heinrich, Massimo Breccia, Michael Deininger, Yeow-Tee Goh, Jeroen J. W. M. Janssen, Moshe Talpaz, Valle Gómez García de Soria, Philipp le Coutre, Francois-Xavier Mahon, Daniel J. DeAngelo, Andrea Damon, Shruti Kapoor, Gessami Sanchez-Olle, Matthias Hoch, Nithya Agrawal, Sara Quenet, Timothy P. Hughes

**Affiliations:** 1 Georgia Cancer Center at Augusta University, Augusta, GA USA; 2 Division of Hematology and Oncology, University of Alabama at Birmingham, Birmingham, AL USA; 3 Department of Hématologie, Hôpital Saint-Louis, Paris, France; 4 Myeloproliferative Neoplasms Program, Memorial Sloan Kettering Cancer Center, New York, NY USA; 5 Hematology/Oncology, Universitätsklinikum Jena, Jena, Germany; 6 Uijeongbu Eulji Medical Center, Geumo-dong, Uijeongbu-si, South Korea; 7 Department of Leukemia, The University of Texas MD Anderson Cancer Center, Houston, TX USA; 8 Department of Laboratory Medicine, Institute of Science Tokyo, Tokyo, Japan; 9 Department of Medicine, Hematology and Oncology, Goethe University Hospital, Frankfurt, Germany; 10 Portland VA Health Care System and OHSU Department of Medicine, Division of Hematology and Oncology, Knight Cancer Institute, Portland, OR USA; 11 Department of Translational and Precision Medicine-Az., Policlinico Umberto I-Sapienza University, Rome, Italy; 12 Versiti Blood Research Institute, Milwaukee, WI USA; 13 Department of Haematology, Singapore General Hospital, Singapore, Singapore; 14 Radboud University Medical Center, Nijmegen, the Netherlands; 15 Division of Hematology-Oncology, University of Michigan Rogel Cancer Center, Ann Arbor, MI USA; 16 Hospital Universitario La Princesa, Madrid, Spain; 17 Department of Oncology and Hematology, Charité - Universitätsmedizin Berlin, Berlin, Germany; 18 Department of Hematology, Institut Bergonié, Bordeaux, France; 19 Dana-Farber Cancer Institute, Boston, MA USA; 20 Novartis Pharmaceuticals Corporation, East Hanover, NJ USA; 21 Novartis Pharma AG, Basel, Switzerland; 22 South Australian Health and Medical Research Institute and University of Adelaide, Adelaide, SA Australia

**Keywords:** Chronic myeloid leukaemia, Chronic myeloid leukaemia, Phase I trials

## Abstract

In chronic myeloid leukemia in chronic phase (CML-CP), *BCR::ABL1*^T315I^ commonly leads to treatment resistance, worse patient outcomes, and limited subsequent treatment options. By targeting the ABL1 myristoyl pocket, asciminib maintains activity against *BCR::ABL1*^T315I^. We report final long-term safety, tolerability, and efficacy results with asciminib in 48 patients with T315I-mutated CML-CP who received asciminib 200 mg twice daily in the phase 1, nonrandomized trial (NCT02081378). After a median exposure of 3.5 years, 52.1% of patients continued to receive asciminib via posttrial access. Of 45 evaluable patients, 24 (53.3%) achieved major molecular response (MMR); 20 of 24 maintained or deepened their response by the cutoff. The Kaplan-Meier estimated proportion of patients maintaining their first MMR for at least 144 weeks (2.8 years) was 86% (95% CI: 71.9–100.0%). The safety profile showed no new or worsening safety signals. With 1.4 years’ additional exposure since the previous analysis, the incidence of grade ≥3 adverse events (AEs) (60.4%) did not increase. Four patients (8.3%) discontinued due to AEs. The exposure-adjusted incidence rate of first all-grade AOEs was 4.4 cases per 100 patient-years. With up to approximately 6 years of exposure, this final analysis confirms asciminib as a treatment option for patients with T315I-mutated CML-CP.

## Introduction

*BCR::ABL1* point mutations are a common mechanism of treatment resistance to tyrosine kinase inhibitors (TKIs) in patients with chronic myeloid leukemia in chronic phase (CML-CP) [1–6]; the T315I mutation (*BCR::ABL1*^T315I^) is among the most common mutations, occurring in up to 22% of patients with resistance to ≥ 1 prior adenosine triphosphate (ATP)–competitive TKI overall [1, 2, 4–7]. Of patients with *BCR::ABL1* mutations and treatment failure, T315I was observed in up to 27% of patients with imatinib as a first TKI and up to 53% with dasatinib or nilotinib as a second TKI [4].

T315I-mutated CML-CP is resistant to most currently approved ATP–competitive TKIs except ponatinib and olverembatinib, limiting treatment options for affected patients [2, 8–12]. Use of these agents may be limited by safety concerns and/or treatment availability; ponatinib carries a risk of cardiovascular (CV) adverse events (AEs), including arterial occlusive events (AOEs), and olverembatinib is currently approved only in China [2, 8–11].

Asciminib, the first approved BCR::ABL1 inhibitor that works by Specifically Targeting the ABL Myristoyl Pocket (STAMP), was designed to improve efficacy and reduce off-target effects compared with ATP–competitive TKIs [13–16]. Asciminib has demonstrated activity against several *BCR::ABL1* mutations that cause resistance to ATP–competitive TKIs, including *BCR::ABL1*^T315I^ [14, 15, 17–19].

The safety and tolerability of asciminib were first established in this first-in-human phase 1 dose-finding trial (cutoff, September 1, 2017; median follow-up, 14 months) [15]. In this trial assessing asciminib up to 200 mg twice daily (BID) in patients with CML-CP—a dose 5-fold higher than that approved for patients without *BCR::ABL1*^T315I^—the maximum tolerated dose of asciminib was not reached [15, 20]. Preclinical observations suggested that inhibition of BCR::ABL1^T315I^ compared with BCR::ABL1 required higher concentrations of asciminib [14, 15, 18, 21–24]. Most patients with *BCR::ABL1*^T315I^ who achieved molecular responses had received asciminib > 150 mg BID, and exposure-response analyses supported the use of asciminib 200 mg BID for T315I-mutated CML-CP [15, 21–24]. Based on these preliminary efficacy and tolerability results, 200 mg BID was chosen as the recommended dose for expansion for T315I-mutated CML-CP [15].

Results from a subsequent analysis of the current phase 1 trial (cutoff, January 6, 2021; median exposure, 2.07 years) further supported the long-term safety and efficacy of asciminib [19]. After up to 4.12 years’ exposure, only 4 patients (8.3%) discontinued treatment due to AEs [19]. Of 45 evaluable patients, 22 (48.9%) achieved major molecular response (MMR), with 19 of 22 having maintained this response by the cutoff [19].

Asciminib 200 mg BID was approved in 2021 in the US for patients with T315I-mutated CML-CP, supported by the current phase 1 trial [15, 19, 20, 24], with subsequent approvals in other countries [25].

We report final efficacy and safety results from the phase 1 study (NCT02081378) in 48 patients with T315I-mutated CML-CP who received asciminib 200 mg BID after ≤6 years of therapy; results in patients with T315I-mutated CML-CP who received asciminib < 200 mg BID are also included.

## Methods

### Study oversight

The study was designed collaboratively by the sponsor (Novartis Pharma AG) and the lead study investigators. The protocol was approved by the sites’ institutional review boards or ethics committees and conducted in accordance with the Declaration of Helsinki. All patients provided written informed consent. Data were collected by the sponsor and analyzed in conjunction with the authors. All authors contributed to the development and writing of the manuscript and vouch for the accuracy and completeness of the data and the study’s fidelity to the protocol.

### Study design

This phase 1, open-label, nonrandomized, first-in-human, dose-finding study has been previously described (Fig. S1) [15, 19, 26]. In study arm 1, adult patients ( ≥ 18 years old) with cytogenetically confirmed Philadelphia chromosome–positive T315I-mutated CML-CP and resistance to or intolerance of ≥ 1 prior TKI (per 2009 European LeukemiaNet recommendations) were enrolled, provided no other effective therapy was available or advisable [15, 27].

The study included an initial dose escalation phase with the primary objective to determine the maximum tolerated dose and/or recommended dose for expansion of asciminib monotherapy [15]. Dose expansion cohorts were opened to further investigate select dose levels with additional endpoints. Safety and tolerability, pharmacokinetics (PK), and preliminary antileukemic activity of asciminib were assessed as secondary objectives [15]. The end of study (EOS) was defined as the time when all enrolled patients had completed study treatment and visits.

A total of 70 patients with T315I-mutated CML-CP were enrolled; 11 received asciminib 20–80 mg BID or 80–200 mg once daily (QD); the remaining patients received asciminib 150 (*n* = 5), 160 (*n* = 6), and 200 (*n* = 48) mg BID in dedicated enrichment cohorts.

This analysis included all 48 patients with CML-CP with a confirmed T315I mutation at screening who received a starting dose of asciminib monotherapy 200 mg BID in the dose-escalation or -expansion phases of this arm of the study. Efficacy data for those who received asciminib < 200 mg BID are also reported.

### Study assessments

Assessment of AEs, molecular response, and *BCR::ABL1* mutations was described previously [15, 26]. *BCR::ABL1* mutational analyses were performed centrally by ICON (Portland, OR, USA) using Sanger sequencing.

### Statistical analyses

The final EOS analysis cutoff was March 14, 2023. Safety analyses included all 48 patients with T315I-mutated CML-CP who received ≥ 1 dose of study drug. Efficacy analyses excluded 3 of 48 patients with atypical or unevaluable *BCR::ABL1* transcripts. Assessment of molecular response rates by time point was defined previously [15, 26]. PK analyses included all patients who provided ≥ 1 blood PK sample.

## Results

### Patients

The primary cohort for this analysis included 48 patients who enrolled from October 2016 to October 2019 with CML-CP with *BCR::ABL1*^T315I^ at baseline and received asciminib 200 mg BID (Table 1 and Fig. S2).

**Table 1:** Disposition by End of Study Cutoff (March 14, 2023).

Patients, *n* (%)	All patients (*n* = 48)
Enrolled	
Treated	48 (100)
Discontinued treatment	23 (47.9)
Reason for discontinuation	
Physician decision due to lack of efficacy^a^	11 (22.9)
Adverse events	4 (8.3)
Progressive disease	2 (4.2)
Patient/guardian decision	2 (4.2)
Death	2 (4.2)
Protocol deviations^b^	1 (2.1)
Technical problems^c^	1 (2.1)
Continued treatment in posttrial access^d^	25 (52.1)

^a^Includes intent to proceed for transplant.

^b^Patient discontinued study treatment due to protocol deviation (use of hydroxyurea for disease control).

^c^Patient could not travel to site due to COVID-19 and discontinued treatment.

^d^Includes but not limited to access to reimbursable commercial supply or the rollover study.

By EOS, 25 patients (52.1%) continued receiving asciminib via posttrial access (PTA), including reimbursable commercial supply or a rollover study. Twenty-three patients (47.9%) discontinued treatment, most frequently due to lack of efficacy (*n* = 11 [22.9%]) and AEs (*n* = 4 [8.3%]). The median duration of exposure was 3.5 years (range, 0.04–6.0 years), including 1.4 years of additional median exposure since the previous analysis [19, 20]. The median dose intensity of asciminib was 398.2 mg/day (range, 171–400 mg/day); most patients (70.8%) received a relative dose intensity of > 90% to 110%.

Most patients (52.1%) had previously received ≥3 TKIs (Table 2) [19]. A majority of patients had received prior dasatinib (68.8%), ponatinib (60.4%), imatinib (56.3%), and/or nilotinib (54.2%) [19]. Patients discontinued their last dose of ponatinib due to resistance (48.3%), intolerance (31.0%), and other reasons (20.7%) [19]. Most ponatinib-pretreated patients (79.3%) had received ≥3 prior TKIs, whereas most ponatinib-naive patients (89.5%) had received 1 or 2 prior TKIs [19]. The distribution of baseline *BCR::ABL1*^IS^ levels was comparable between ponatinib-pretreated and -naive patients. Two patients had additional baseline *BCR::ABL1* mutations (E255K, E355G [*n* = 1 each]) [19].

**Table 2:** Patient Demographics and Baseline Characteristics.

Demographic variable	All patients *n* = 48	Evaluable patients *n* = 45
Age, median (range), years	56.5 (26–86)	54.0 (26–86)
Age category, *n* (%)		
18 to < 65 years	32 (66.7)	31 (68.9)
≥65 years	16 (33.3)	14 (31.1)
≥75 years	4 (8.3)	4 (8.9)
Sex, *n* (%)		
Female	11 (22.9)	9 (20.0)
Male	37 (77.1)	36 (80.0)
Race, *n* (%)		
Asian	12 (25.0)	12 (26.7)
Black or African American	1 (2.1)	1 (2.2)
Other	6 (12.5)	6 (13.3)
Unknown	6 (12.5)	5 (11.1)
White	23 (47.9)	21 (46.7)
Ethnicity, *n* (%)		
East Asian	10 (20.8)	10 (22.2)
Hispanic or Latino	3 (6.3)	3 (6.7)
Not reported	14 (29.2)	13 (28.9)
Other	16 (33.3)	14 (31.1)
Southeast Asian	2 (4.2)	2 (4.4)
Unknown	3 (6.3)	3 (6.7)
ECOG performance status, *n* (%)		
0	36 (75.0)	33 (73.3)
1	12 (25.0)	12 (26.7)
No. of prior TKIs^a^		
1^b^	8 (16.7)	8 (17.8)
2	15 (31.3)	14 (31.1)
3	17 (35.4)	16 (35.6)
≥4	8 (16.7)	7 (15.6)
Individual prior TKIs		
Bosutinib	3 (6.3)	2 (4.4)
Dasatinib	33 (68.8)	33 (73.3)
Imatinib	27 (56.3)	25 (55.6)
Nilotinib	26 (54.2)	23 (51.1)
Ponatinib	29 (60.4)	26 (57.8)
Radotinib	4 (8.3)	4 (8.9)
Reason for discontinuation of the last dose of ponatinib		
Intolerance	9 (31.0)	7 (26.9)
Resistance	14 (48.3)	13 (50.0)
Other^c^	6 (20.7)	6 (23.1)
Mutations at screening, *n* (%)		
T315I alone	46 (95.8)	43 (95.6)
T315I and E255K	1 (2.1)	1 (2.2)
T315I and E355G	1 (2.1)	1 (2.2)
*BCR::ABL1*^IS^ at screening, *n* (%)^d^		
>0.01% to 0.1%	0	0
>0.1% to 1%	8 (16.7)	8 (17.8)^d^
>1% to 10%	11 (22.9)	11 (24.4)^e^
>10%	26 (54.2)	26 (57.8)^f^
Atypical/e1a2/unknown transcripts^g^	3 (6.3)	0

Reprinted from Cortes JE, et al. *Leukemia*. 2024;38(7):1522–1533.(19) Creative Commons Attribution 4.0 International License (https://creativecommons.org/licenses/by/4.0/).

ECOG, Eastern Cooperative Oncology Group.

^a^Of 29 ponatinib-pretreated patients, 6 (20.7%), 15 (51.7%), and 8 (27.6%) received 2, 3, and ≥4 prior TKIs, respectively. Of 19 ponatinib-naive patients, 8 (42.1%), 9 (47.4%), and 2 (10.5%) received 1, 2, and 3 prior TKIs, respectively.

^b^Prior TKIs included dasatinib (*n* = 5), nilotinib (*n* = 2), and radotinib (*n* = 1).

^c^Other includes enrollment in this phase 1 trial (*n* = 4) and completion of prescribing regimen (*n* = 2).

^d^Included 5 of 26 ponatinib-pretreated patients (19.2%) and 3 of 19 ponatinib-naive patients (15.8%).

^e^Included 6 of 26 ponatinib-pretreated patients (23.1%) and 5 of 19 ponatinib-naive patients (26.3%).

^f^Included 15 of 26 ponatinib-pretreated patients (57.7%) and 11 of 19 ponatinib-naive patients (57.9%).

^g^e19a2 transcript, e13a3 (b2a3) transcript, no transcript detected (*n* = 1, each).

### Efficacy

#### Patients who received a starting dose of asciminib 200 mg BID

**Fig. 1:**
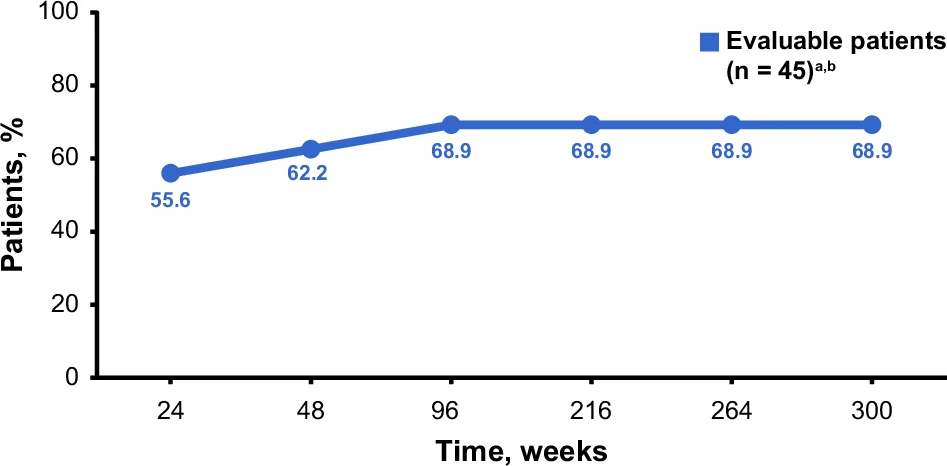
Cumulative rate of *BCR::ABL1*^IS^ ≤ 1% in all molecular response–evaluable patients, including those with response at baseline. Reused with permission from the American Society of Hematology. © 2024 The Authors. All rights reserved. Officially licensed by ASH for distribution via Novartis Pharmaceuticals. ^a^3 patients were excluded from efficacy analyses due to atypical/e1a2/e1a3/unknown *BCR::ABL1* transcripts. ^b^8 of 45 patients had *BCR::ABL1*^IS^ ≤ 1% at baseline.

Of 48 total patients, 3 were excluded from all molecular response analyses due to atypical (*n* = 2) or no (*n* = 1) *BCR::ABL1* transcripts detected at baseline [19]. Most patients (25 [55.6%]) had *BCR::ABL1*^IS^ ≤ 1% by week 24, with new patients continuing to achieve a first response up to week 96 (1.8 years; Fig. 1). By week 300 (5.8 years), 31 patients (68.9%) had *BCR::ABL1*^IS^ ≤ 1%; 8 had this response at baseline, and 23 achieved this response. The Kaplan-Meier estimated proportion of patients maintaining first *BCR::ABL1*^IS^ ≤ 1% for at least 144 weeks (2.8 years) in patients who achieved this response was 80% (95% CI: 63.0–97.7%).

**Fig. 2:**
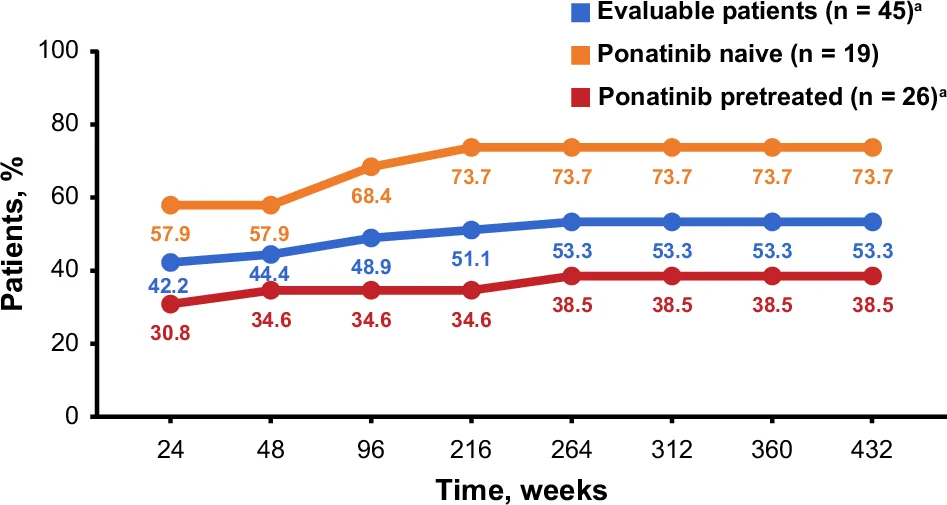
Cumulative MMR rate in molecular response–evaluable patients not in MMR at screening. Reused with permission from the American Society of Hematology. © 2024 The Authors. All rights reserved. Officially licensed by ASH for distribution via Novartis Pharmaceuticals. MMR, major molecular response (*BCR::ABL1* ≤ 0.1% on the International Scale). ^a^3 patients were excluded from efficacy analyses due to atypical/e1a2/e1a3/unknown *BCR::ABL1* transcripts.

By EOS, MMR was achieved in 24 patients (53.3%), including 2 additional patients since the previous analysis (Fig. 2) [19]. Most patients (*n* = 20) had achieved MMR by week 24; most patients (80%) with ≥24 weeks of treatment were receiving asciminib 200 mg BID at this time [19]. Patients continued to achieve first MMR up to week 264 (5.1 years). The median time to MMR was 16.2 weeks (range, 4–264 weeks). Responses were durable; 20 of 24 patients who achieved MMR maintained or deepened their response by EOS. Four patients lost MMR; 1 of these lost response after the previous analysis [19]. After loss of MMR, 1 patient regained MMR and discontinued treatment due to technical problems (inability to travel to site due to COVID-19) and continued receiving asciminib in PTA; 1 patient maintained *BCR::ABL1*^IS^ ≤ 1% and continued receiving asciminib in PTA; the remaining 2 patients had *BCR::ABL1*^IS^ ≤ 10% and discontinued treatment due to physician decision (elected to undergo hematopoietic stem cell transplant and patient/guardian decision [*n* = 1 each]). No association between dose adjustment and loss of MMR was observed. The Kaplan-Meier estimated proportion of patients maintaining first MMR for at least 144 weeks (2.8 years) was 86% (95% CI: 71.9–100.0%). The MMR rate at time points remained relatively stable ( ± 5%) from weeks 24 (37.8%) to 96 (35.6%) (Table S1). Neither of the 2 patients with additional baseline mutations achieved MMR: the patient with baseline E255K achieved *BCR::ABL1*^IS^ ≤ 1% by week 12 and discontinued due to the patient’s decision to try other treatments; the patient with baseline E355G had *BCR::ABL1*^IS^ ≥ 10% and discontinued due to the physician’s decision to transition to an investigator-initiated trial.

The cumulative MMR rate remained consistently higher over time in ponatinib-naive vs -pretreated patients; by EOS, MMR was achieved in 14 of 19 evaluable ponatinib-naive (73.7%) and 10 of 26 evaluable ponatinib-pretreated patients (38.5%). Median time to MMR was 20.1 weeks (range, 4–156 weeks) in ponatinib-naive and 12.1 weeks (range, 4–264 weeks) in ponatinib-pretreated patients. Of the 4 patients who lost MMR, 3 had prior exposure to 3 TKIs, including ponatinib; 1 ponatinib-naive patient had prior exposure to 2 TKIs. The Kaplan-Meier estimated proportion of patients maintaining first MMR for at least 144 weeks was 92% (95% CI: 76.0–100.0%) in ponatinib-naive and 79% (95% CI: 52.5–100.0%) in ponatinib-pretreated patients.

**Fig. 3:**
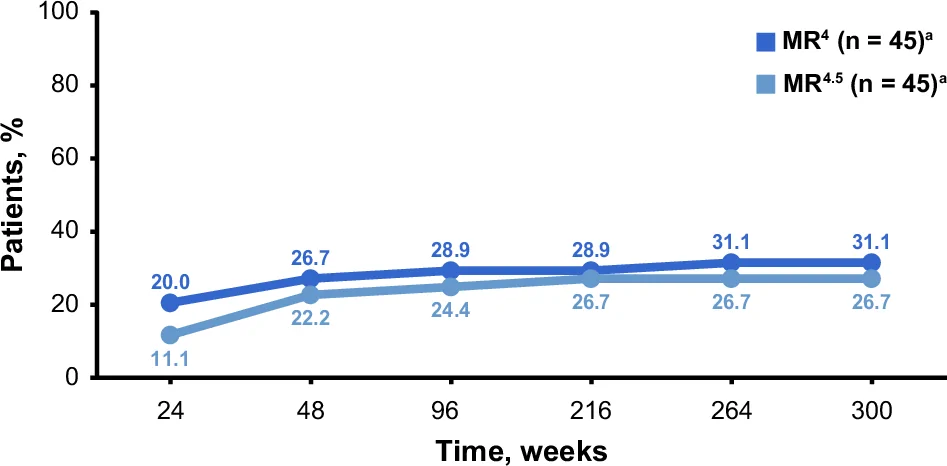
Cumulative rate of deep molecular response (DMR [MR^4^ or better]) in molecular response–evaluable patients without DMR at screening. Reused with permission from the American Society of Hematology. © 2024 The Authors. All rights reserved. Officially licensed by ASH for distribution via Novartis Pharmaceuticals. IS, International Scale; MR^4^, *BCR::ABL1*^IS^ ≤ 0.01%; MR^4.5^, *BCR::ABL1*^IS^ ≤ 0.0032%. ^a^3 patients were excluded from efficacy analyses due to atypical/e1a2/e1a3/unknown *BCR::ABL1* transcripts.

In the 45 evaluable patients, deep molecular responses (DMRs; MR^4^ or better) were also observed; 14 patients (31.1%) achieved at least MR^4^, and 12 (26.7%) achieved MR^4.5^ (Fig. 3). New DMRs continued to be achieved up to week 264 (5.1 years), including in 1 new patient since the previous analysis [19].

#### Patients who received a starting dose of asciminib < 200 mg BID

By EOS, 2 of 5 and 1 of 6 patients who received asciminib 150 and 160 mg BID, respectively, continued to receive asciminib in PTA (Table S2). Most patients (63.6%) received a relative dose intensity of > 90% to 110% (Table S3). One patient in each of these cohorts was excluded from efficacy analyses due to atypical/e1a2/e1a3/unknown *BCR::ABL1* transcripts. Overall, of evaluable patients who received 150 (*n* = 4) and 160 (*n* = 5) mg BID, respectively, 2 and 3 achieved or maintained *BCR:ABL1*^IS^ ≤ 1%, and 2 and 2 achieved or maintained MMR (Fig. S3); 1 patient in each cohort had a best response of MR^4.5^. In patients who received asciminib at all other dose levels ( ≤ 80 mg BID and ≤ 200 mg QD; *n* = 11), the best responses achieved included *BCR::ABL1*^IS^ ≤ 1% (*n* = 2; both received 80 mg BID) and MR^4.5^ (*n* = 1; received 20 mg QD).

### Safety

The safety profile of asciminib was consistent with previous analyses. In the current analysis, no new or worsening safety signals were observed in the asciminib safety profile. The most common ( ≥ 25%) all-grade AEs included fatigue (31.3%), increased lipase (29.2%), and diarrhea and nausea (27.1% each; Table 3). Most AEs occurred in the first year of treatment, and few patients experienced a first-ever event after the first year (Fig. S4). Since the previous analysis, the rate of grade ≥3 AEs (60.4%) and AEs leading to discontinuation (10.4%) did not increase (Table S4) [19]. The most common ( ≥ 10%) grade ≥3 events included increased lipase (20.8%), thrombocytopenia (16.7%), and neutropenia (12.5%). COVID-19 pneumonia (*n* = 2) was the most frequent AE leading to treatment discontinuation. AEs led to dose adjustment and/or interruption in 21 patients (43.8%). AEs that led to dose adjustment and/or interruption were suspected to be treatment related in 15 patients (31.3%); the most frequent ( > 5%) were increased lipase (14.6%) and thrombocytopenia (6.3%) (Table S5). AEs required additional therapy in 39 patients (81.3%).

**Table 3:** Adverse Events, Regardless of Relationship to Study Treatment, that Occurred in ≥ 10% of Patients.

	All patients (*n* = 48)	
Preferred term, *n* (%)^a,b^	All grades	Grade ≥ 3
Patients with ≥1 event	48 (100)	29 (60.4)
Fatigue	15 (31.3)	1 (2.1)
Lipase increased	14 (29.2)	10 (20.8)
Diarrhea	13 (27.1)	1 (2.1)
Nausea	13 (27.1)	0
Cough	11 (22.9)	0
Arthralgia	10 (20.8)	0
Headache	10 (20.8)	1 (2.1)
Alanine aminotransferase increased	10 (20.8)	4 (8.3)
Vomiting	10 (20.8)	3 (6.3)
Thrombocytopenia^c^	10 (20.8)	8 (16.7)
Musculoskeletal pain^d^	10 (20.8)	0
Neutropenia^e^	8 (16.7)	6 (12.5)
Aspartate aminotransferase increased	7 (14.6)	1 (2.1)
Hypertension	7 (14.6)	4 (8.3)
Back pain	7 (14.6)	1 (2.1)
Abdominal pain	7 (14.6)	3 (6.3)
Rash	6 (12.5)	0
Pyrexia	6 (12.5)	0
Constipation	6 (12.5)	0
Pruritus	6 (12.5)	0
Hyperuricemia	6 (12.5)	0
Amylase increased	6 (12.5)	2 (4.2)
Anemia	5 (10.4)	3 (6.3)
Noncardiac chest pain	5 (10.4)	1 (2.1)
Myalgia	4 (8.3)	0
Dizziness	4 (8.3)	0
γ-glutamyltransferase increased	4 (8.3)	2 (4.2)
Hypophosphatemia	3 (6.3)	1 (2.1)
Dyspnea	3 (6.3)	0

Reused with permission from the American Society of Hematology. © 2024 The Authors. All rights reserved. Officially licensed by ASH for distribution via Novartis Pharmaceuticals.

^a^A patient with multiple severity grades for an adverse event is only counted under the maximum grade.

^b^COVID-19 is not reported in this table.

^c^Includes thrombocytopenia and decreased platelet count.

^d^Include musculoskeletal pain and pain in extremity.

^e^Includes neutropenia and a decreased neutrophil count.

When AEs were grouped by clinically important safety categories, the most frequent AEs of special interest (AESIs) were gastrointestinal toxicity (50.0%), hypersensitivity (33.3%), pancreatic toxicity (clinical pancreatitis and isolated pancreatic enzyme elevations; 31.3%), and hepatotoxicity (including laboratory terms; 31.3%) (Table 4). A majority of the most frequent AESIs were resolved or are resolving at EOS. The exposure-adjusted incidence rates for all-grade AESIs are reported in Table S6.

**Table 4:** Adverse Events of Special Interest.

	All patients (*n* = 48)	
Patients, *n* (%)^a^	All grades	Grade ≥ 3
Gastrointestinal toxicity	24 (50.0)	5 (10.4)
Hypersensitivity	16 (33.3)	1 (2.1)
Pancreatic toxicity (including clinical pancreatitis and isolated pancreatic enzyme elevations)	15 (31.3)^b^	11 (22.9)
Pancreatitis (clinical events)	1 (2.1)^c^	0
Hepatotoxicity (including laboratory terms)	15 (31.3)	6 (12.5)
Myelosuppression	14 (29.2)	10 (20.8)
Erythropenia (anemia)	5 (10.4)	3 (6.3)
Neutropenia	8 (16.7)	6 (12.5)
Thrombocytopenia	10 (20.8)	8 (16.7)
Cytopenias affecting > 1 lineage	1 (2.1)	1 (2.1)
Hemorrhage	10 (20.8)	1 (2.1)
Edema and fluid retention	9 (18.8)	3 (6.3)
Ischemic heart and CNS conditions	7 (14.6)	3 (6.3)
Ischemic heart disease	5 (10.4)	2 (4.2)
Ischemic CNS vascular conditions	4 (8.3)	2 (4.2)
Cardiac failure (clinical events)	1 (2.1)	1 (2.1)
Phototoxicity	1 (2.1)	0
QTc prolongation	1 (2.1)	1 (2.1)

Reused with permission from the American Society of Hematology. © 2024 The Authors. All rights reserved. Officially licensed by ASH for distribution via Novartis Pharmaceuticals.

CNS, central nervous system; QTc, corrected QT interval.

^a^A patient with multiple severity grades for an AE is only counted under the maximum grade.

^b^Events included lipase increased (14 [29.2%]), amylase increased (6 [12.5%]), hyperlipasemia (1 [2.1%]), and pancreatitis (1 [2.1%])

^c^One patient experienced pancreatitis (grade 2), which did not require a treatment change.

The most frequent ( ≥ 10%) gastrointestinal events were nausea and diarrhea (27.1% each), vomiting (20.8%), abdominal pain (14.6%), constipation (12.5%), and noncardiac chest pain (10.4%); grade 3 events occurred in 5 patients (10.4%); no grade 4 events occurred. Dose adjustment and interruption for gastrointestinal events were required in 2 (4.2%) and 1 (2.1%) patients, respectively. Rash (12.5%) was the most frequent ( ≥ 10%) hypersensitivity event. One patient experienced a grade 3 hypersensitivity event (iodine allergy). None of these events required dose adjustment and/or interruption or led to treatment discontinuation. The most frequent ( ≥ 10%) pancreatic toxicity events were increased lipase (29.2%) and amylase (12.5%). Grade ≥3 events occurred in 11 patients (22.9%). Dose adjustment and interruption were required in 5 (10.4%) and 8 (16.7%) patients, respectively. As previously described, clinical pancreatitis (grade 2) occurred in 1 patient, who required concomitant medication and was resolving at EOS [19]. The most frequent ( ≥ 10%) hepatotoxicity events were increased alanine aminotransferase (20.8%) and aspartate aminotransferase (14.6%). Grade 3 events occurred in 6 patients (12.5%); no grade 4 events occurred.

AOEs occurred in 6 patients (12.5%) (Table S7) [19]. When adjusted for patient-year exposure, the incidence rate of first all-grade AOEs was 4.4 cases per 100 patient-years. Grade 3 and 4 events occurred in 2 (4.2%) and 1 patients (2.1%), respectively. Since the previous analysis, 2 additional patients experienced AOEs. One patient experienced grade 2 acute coronary syndrome and continued to receive asciminib in PTA. One patient experienced grade 2 peripheral arterial occlusive disease, grade 2 ischemic stroke, and grade 3 ischemic stroke while on study; 24 days after having discontinued the study and continuing to receive asciminib in PTA, they experienced cerebrovascular accident and myocardial infarction (both grade 4), which resulted in death. Of the 6 patients who experienced AOEs, 4 completed the trial and continued to receive asciminib in PTA. Narratives for all patients with AOEs are listed in Table S8. No AOEs led to dose adjustment, interruption, or discontinuation of study treatment.

Three patients (6.3%) died on study; 2 of these died on treatment or within 30 days of the last study drug dose. As previously reported, 1 patient died off treatment ( > 30 days after having discontinued study treatment) due to pneumonia, with COVID-19 as a contributing factor; 1 patient died on treatment due to COVID-19 pneumonia [19]. The newly reported death occurred on treatment, 24 days after the patient had completed the trial and continued receiving asciminib in PTA, due to cerebrovascular accident and myocardial infarction (Supplemental material).

### Pharmacokinetics

Table S9 summarizes PK parameters in patients with T315I-mutated CML-CP receiving asciminib 200 mg BID. No apparent differences were observed in the PK parameters of patients with CML-CP with or without *BCR::ABL1*^T315I^ [28].

Based on previously reported safety, tolerability, and preliminary efficacy results from this trial, asciminib 200 mg BID was chosen as the recommended dose for expansion in patients with T315I-mutated CML-CP [15].

## Discussion

This updated, final analysis with up to ≈6 years of exposure in the phase 1 study is the longest follow-up of asciminib in patients with T315I-mutated CML-CP. After additional exposure, asciminib continued to exhibit high clinical efficacy and sustained safety and tolerability in T315I-mutated CML-CP [19]. Asciminib was well tolerated by most patients, with low rates of discontinuation due to AEs, and over half of all patients continued asciminib in PTA.

The efficacy results observed with asciminib 200 mg BID, compared with 150 or 160 mg BID, continue to support asciminib 200 mg BID for T315I-mutated CML-CP [14, 15, 18, 21–24]. Patients continued to benefit from asciminib over time, with additional patients achieving a first molecular response late in treatment (1.8 to 5.1 years). International recommendations lack guidance regarding optimal response milestones for T315I-mutated CML-CP; however, a response of *BCR::ABL1*^IS^ > 1% is not considered sufficient to improve survival [2, 12, 29]. *BCR::ABL1*^IS^ ≤ 1% is associated with an increased overall survival rate and a decreased disease progression rate [12, 30–33]. A clinically meaningful proportion of patients (68.9%) achieved or maintained this response by EOS and were likely to maintain it for ≥ 2.8 years.

Most patients (53.3%), including those with and without prior ponatinib exposure, achieved MMR. Responses were durable; many patients achieved MMR within the first year of treatment, and almost all (20 of 24) who achieved MMR maintained or improved this response by EOS. The MMR rates in this study agree with those in a real-world evidence study including 31 patients with T315I-mutated CML treated with asciminib from 8 countries; 40.0% and 41.7% of evaluable patients achieved MMR by 6 and 12 months, respectively [34]. When considered alongside trial results for other therapies for T315I-mutated CML-CP, MMR rates observed in the current study are numerically higher. For context, in the PACE trial assessing ponatinib 45 mg QD, 58% of patients with T315I-mutated CML-CP after ≥ 2 prior TKIs achieved MMR by 4.73 years, with 60% of those who achieved MMR estimated to maintain it at 5 years [10]. In the OPTIC trial, which assessed ponatinib starting doses of 45, 30, and 15 mg QD, respectively, 34.4%, 24.7%, and 23.1% of patients with CML-CP with or without *BCR::ABL1*^T315I^ after ≥ 2 prior TKIs had MMR at cutoff (32 months’ median follow-up) [9]. In a phase 1 trial (48 weeks’ median follow-up) that assessed olverembatinib 50, 40, and 30 mg every other day, 44.4% of patients with T315I-mutated CML after ≥ 2 prior TKIs achieved MMR [35]. Although cross-trial comparisons should be interpreted cautiously, these data suggest that asciminib provides meaningful and durable responses in patients with T315I-mutated CML-CP.

The most common AEs in this population were consistent with those of the primary analysis (cutoff, April 2020), with no new or worsening safety findings in the asciminib safety profile observed with longer asciminib exposure. Most first-ever AEs occurred early, in the first year of treatment, and overall, most AEs were low-grade (1/2).

The safety profile of asciminib 200 mg BID was similar to that of 40 mg BID and 80 mg QD in the current phase 1 study and 40 mg BID in ASCEMBL, indicating a large therapeutic window [28, 36]. There were no new or worsening safety findings compared with the 40 mg BID dose recommended for patients without *BCR::ABL1*^T315I^ in this trial. Analysis of 353 patients with ≥1 prior TKIs from the phase 1 study receiving asciminib 10–200 mg BID or 80–200 mg QD and with ≥ 2 prior TKIs from ASCEMBL receiving asciminib 40 mg BID found no clinically relevant relationship between exposure and the rate of AEs [22]; an updated analysis of 550 patients, which included additional patients with newly diagnosed CML-CP from the ASC4FIRST trial, reported no clinically relevant relationship between exposure and the rate of most AEs. In this updated analysis, the probability of safety events was higher in patients with ≥2 prior TKIs compared with those who were newly diagnosed overall [37].

Most AESIs were resolved or resolving (improved or improving in toxicity grade) at EOS. Pancreatic toxicity events comprised mainly enzyme elevations and infrequently required dose adjustment and/or interruption. As reported previously, 1 patient experienced grade 2 clinical pancreatitis [19]. This event did not require a treatment change and was resolving at EOS. With ≤ 8.4 years’ exposure in patients with CML-CP without *BCR::ABL1*^T315I^ who received asciminib 10–200 mg BID or 80–200 mg QD in the phase 1 trial, 8 (7.0%) experienced clinical pancreatitis; no grade 4 events occurred, and only 2 events led to treatment discontinuation [28]. With 3.7 years’ median follow-up in ASCEMBL, no cases of clinical pancreatitis were reported, and few patients who received asciminib experienced grade ≥ 3 enzyme elevations (lipase, 3.8%; amylase, 0.6%) [36].

Treatment with TKIs has been associated with CV risk, specifically AOEs [38, 39]. The exposure-adjusted incidence rate of all-grade AOEs (4.4%) was consistent with that of the previous analysis (4.3%) [19]. No AOE led to dose adjustment/interruption/discontinuation. While no grade 4 AOEs were reported previously, 2 grade 4 events (cerebrovascular accident and myocardial infarction) occurred in 1 patient since the last cutoff and led to death [19]. This on-treatment death was the only additional study death reported since the previous analysis [19]. The patient had received 4 prior TKIs and had multiple CV risk factors, including past acute coronary syndrome, dyslipidemia, stent placement, coronary angioplasty, and myocardial infarction, and ongoing hypertension, hyperglycemia, myocardial necrosis, and tachycardia at screening. Five of 6 patients with AOEs were ≥52-year-old men. All 6 patients had ≥1 past or active CV risk factor at baseline and prior exposure to 2 (*n* = 1), 3 (*n* = 2), or 4 TKIs (*n* = 3); individual prior TKIs included imatinib (*n* = 5), ponatinib (*n* = 5), nilotinib (*n* = 5), dasatinib (*n* = 3), and bosutinib (*n* = 2). Due to multiple confounding factors, the role of asciminib in the development or exacerbation of these events remains uncertain [40–42]. After up to 8.4 years’ exposure in patients with CML-CP without *BCR::ABL1*^T315I^ in the current phase 1 trial, the exposure-adjusted incidence rate of AOEs was 2.6% [28]. With 3.7 years’ median follow-up in ASCEMBL, the exposure-adjusted incidence rate of AOEs (2.2%) decreased from the primary analysis (3.3%) [36, 43]. In OPTIC, after 32 months’ median follow-up, AOEs occurred in 9.6%, 5.3%, and 3.2% of patients receiving ponatinib 45, 30, and 15 mg, respectively [9]. Of patients with CML and acute lymphoblastic leukemia in the phase 1 trial of olverembatinib, AOEs occurred in 9.1%, 15.6%, and 8.1% receiving olverembatinib 50, 40, and 30 mg, respectively [35]. The rates of AOEs observed across asciminib trials in pretreated CML-CP support the favorable safety of long-term asciminib treatment; however, patients receiving TKIs–especially those with a history of CV risk factors–should be assessed for baseline risk and actively monitored and treated as clinically indicated [20, 38, 39].

Hypertension is associated with TKI use [10, 12, 36, 44–46]. In this study, all-grade hypertension rates (all grade, 14.6%; grade ≥3, 8.3%) were numerically lower than those observed in patients with CML-CP with ponatinib in OPTIC (all grade, 23% to 34%; grade ≥3, 5% to 10%) [9] and similar to those in patients with CML and acute lymphoblastic leukemia who received olverembatinib (all grade, 3.1% to 16.2%; grade ≥3, 0% to 10.8%), dependent on dose [35]. In patients receiving asciminib, blood pressure should be monitored, with hypertension managed as clinically indicated [20].

In the current phase 1 study, the MMR rate remained consistently higher in ponatinib-naive (73.7%) than ponatinib-pretreated patients (38.5%). This observation is supported by several real-world evidence studies, including those in patients without *BCR::ABL1*^T315I^ [47–55]. In the previous analysis with 2.07 years’ asciminib exposure, overall MMR rates in ponatinib-pretreated patients were higher in those who had discontinued ponatinib due to intolerance (57.1%) compared with resistance (15.4%) [19]. This observation is supported by real-world evidence studies of ponatinib-pretreated patients with or without *BCR::ABL1*^T315I^, while one study found that the MMR rate was comparable when analyzing all patients who discontinued their prior treatment due to resistance or intolerance [48–50, 52, 54, 55]. Additionally, in the phase 1 trial of olverembatinib, MMR rates were higher in patients with T315I-mutated CML who received prior ponatinib (46.2%) compared with prior asciminib (33.3%) [35]. The increased efficacy of asciminib in ponatinib-naive vs -pretreated patients, together with its excellent safety profile, supports its use prior to ponatinib for T315I-mutated CML-CP. Per the 2025 European LeukemiaNet recommendations, eligible patients with resistance to ponatinib or asciminib should be considered for allogeneic transplant [12].

Asciminib continued to exhibit a high rate of sustained efficacy and consistent safety and tolerability in patients with T315I-mutated CML-CP—a population with poor long-term outcomes and limited treatment options. Durable MMR was observed in ponatinib-naive and -pretreated patients. Remarkably, after up to ≈6 years’ exposure, over half of patients continued treatment with asciminib in PTA, and only 8.3% discontinued due to AEs. This final analysis of the phase 1 trial confirms asciminib as an efficacious and tolerable treatment for patients with T315I-mutated CML-CP.

## Supplementary information

Supplemental Appendix

## Data Availability

Novartis is committed to sharing with qualified external researchers access to patient-level data and supporting clinical documents from eligible studies. These requests are reviewed and approved by an independent review panel on the basis of scientific merit. All data provided are anonymized to respect the privacy of patients who have participated in the trial in line with applicable laws and regulations. The trial data availability is according to the criteria and process described on http://www.clinicalstudydatarequest.com.
