# Supplementary material for: Asciminib monotherapy in patients with BCR::ABL1 T315I-mutated chronic-phase chronic myeloid leukemia: phase 1 trial final results
Source: Leukemia. 2026 Jul 16;40(9):1893–900. doi: 10.1038/s41375-026-02972-9 (PMC13506337; doi:10.1038/s41375-026-02972-9)
Supplement: Supplementary file 1 [file 41375_2026_2972_MOESM1_ESM.docx]

**SUPPLEMENTAL RESULTS**

**Newly reported on-treatment (within 30 days of the last study drug dose) death**

- A 73-year-old female patient was enrolled in the study after 4 prior TKIs (imatinib, nilotinib, ponatinib, and bosutinib).
  - Relevant past medical conditions included skin neoplasm excision, hysterectomy, acute coronary syndrome, dyslipidemia, stent placement, ischemic heart disease prophylaxis, coronary angioplasty, myocardial infarction, and tendon disorder.
  - Relevant active medical conditions included hypertension, hyperglycemia, overweight, myocardial necrosis, and tachycardia.
  - On study day 625, the patient experienced peripheral arterial occlusive disease (grade 2).
  - The patient later experienced 2 episodes of ischemic stroke (grade 2 [day 980] and grade 3 [day 988]).
  - No action was taken with asciminib for arterial occlusive disease or ischemic stroke.
  - On study day 1092, the patient completed the trial and continued to receive asciminib in posttrial access.
  - Cerebrovascular accident (grade 4) and myocardial infarction (grade 4) occurred on day 1116 and resulted in death; peripheral arterial occlusive disease was ongoing at the time of death.
  - The investigator did not suspect a relationship between ischemia stroke (both episodes), cerebrovascular accident, myocardial infarction, and peripheral arterial occlusive disease and study treatment.

**Table S1:** Major Molecular Response Rate at Time Points in Evaluable Patients by Ponatinib Pretreatment Status

| **Time point, n (%)^a^** | **Ponatinib naive**  **(n = 19)** | **Ponatinib pretreated**  **(n = 26)** | **All patients**  **(n = 45)** |
| --- | --- | --- | --- |
| At week 24 | 10 (52.6) | 7 (26.9) | 17 (37.8) |
| At week 48 | 9 (47.4) | 7 (26.9) | 16 (35.6) |
| At week 96 | 11 (57.9) | 5 (19.2) | 16 (35.6) |
| At week 156 | 9 (47.4) | 4 (15.4) | 13 (28.9) |
| At week 204 | 9 (47.4) | 3 (11.5) | 12 (26.7) |
| At week 276 | 1 (5.3) | 2 (7.7) | 3 (6.7) |

^a^ Responders with missing intermediate visits, for weeks other than weeks 24 and 48, were considered as nonresponders (not having maintained their response) for that visit.

**Table S2:** Disposition of Patients Who Received Asciminib < 200 mg Twice Daily

| **Patients, n (%)** | **Asciminib 80-200 mg QD and 20-80 mg BID**  **(n = 11)^a^** | **Asciminib 150 mg BID**  **(n = 5)** | **Asciminib 160 mg BID**  **(n = 6)** |
| --- | --- | --- | --- |
| Discontinued treatment | 10 (90.9) | 3 (60.0) | 5 (83.3) |
| Reason for discontinuation |  |  |  |
| Patient decision | 5 (45.5) | 0 | 0 |
| Physician decision due to lack of efficacy^b^ | 3 (27.3) | 1 (20.0) | 3 (50.0) |
| Adverse events | 1 (9.1) | 1 (20.0) | 0 |
| Death | 1 (9.1) | 0 | 0 |
| Progressive disease | 0 | 1 (20.0) | 1 (16.7) |
| Technical problems | 0 | 0 | 1 (16.7) |
| Continued treatment in posttrial access^c^ | 1 (9.1) | 2 (40.0) | 1 (16.7) |

Reused with permission from the American Society of Hematology. © 2024 The Authors. All rights reserved. Officially licensed by ASH for distribution via Novartis Pharmaceuticals.

BID, twice daily; QD, once daily.

^a^ Includes patients who received starting doses of asciminib at 80 (n = 1), 120 (n = 3), and 200 (n = 1) mg QD and 20 (n = 1), 40 (n = 1), and 80 (n = 4) mg BID.

^b^ Includes intent to proceed for transplant.

^c^ Includes but not limited to access to reimbursable commercial supply or the rollover study.

**Table S3:** Dose Intensity of Patients Who Received Asciminib 150 or 160 mg BID

| **Patients, n (%)** | **Asciminib 150 mg BID**  **(n = 5)** | **Asciminib 160 mg BID**  **(n = 6)** |
| --- | --- | --- |
| Dose intensity, median (range), mg/day | 317.6  (300-356) | 320.0  (182-365) |
| **Relative dose intensity categories** |  |  |
| ≤ 75% | 0 | 1 (6.7) |
| > 75% to 90% | 0 | 0 |
| > 90% to 100% | 4 (80.0) | 3 (50.0) |
| > 110% | 1 (20.0) | 2 (33.3) |

BID, twice daily.

**Table S4:** Adverse Events Leading to Discontinuation

| **Preferred term, n (%)** | **All patients**  **(n = 48)** |
| --- | --- |
| Patients with ≥ 1 event | 5 (10.4) |
| COVID-19 pneumonia | 2 (4.2) |
| Lipase increased | 1 (2.1) |
| Neutropenia^a,b^ | 1 (2.1) |
| Pancytopenia^a^ | 1 (2.1) |
| Thrombocytopenia^a,c^ | 1 (2.1) |
| Thrombocytosis | 1 (2.1) |

Adapted from Cortes JE, et al. *Leukemia*. 2024;38(7):1522-1533.(1) Creative Commons Attribution 4.0 International License (<https://creativecommons.org/licenses/by/4.0/>).

^a^ Occurred in the same patient. In the previous analysis, this patient was listed as discontinuing due to pancytopenia alone.

^b^ Includes neutropenia and neutrophil count decreased.

^c^ Includes thrombocytopenia and platelet count decreased.

**Table S5:** Adverse Events Leading to Treatment Adjustment and/or Interruption Suspected to be Related to Study Treatment

| **Preferred term, n (%)^a^** | **All patients**  **(n = 48)** |
| --- | --- |
| Patients with ≥ 1 event | 15 (31.3) |
| Lipase increased | 7 (14.6) |
| Thrombocytopenia | 3 (6.3) |
| Neutropenia | 2 (4.2) |
| Amylase increased | 2 (4.2) |
| Anemia | 1 (2.1) |
| Abdominal pain | 1 (2.1) |
| Nausea | 1 (2.1) |
| Fatigue | 1 (2.1) |
| Malaise | 1 (2.1) |
| Alanine aminotransferase increased | 1 (2.1) |
| Blood bilirubin increased | 1 (2.1) |
| Blood creatinine increased | 1 (2.1) |
| γ-Glutamyltransferase increased | 1 (2.1) |
| Decreased appetite | 1 (2.1) |
| Dehydration | 1 (2.1) |
| Hyperkalemia | 1 (2.1) |
| Hyperlipasemia | 1 (2.1) |
| Arthralgia | 1 (2.1) |
| Back pain | 1 (2.1) |
| Musculoskeletal pain | 1 (2.1) |
| Pain in extremity | 1 (2.1) |
| Scleroderma | 1 (2.1) |
| Dizziness | 1 (2.1) |
| Pruritus | 1 (2.1) |

^a^ A patient with multiple severity grades for an adverse event is only counted under the maximum grade.

**Table S6:** Exposure-Adjusted Incidence Rates of Adverse Events of Special Interest

|  | **All patients (n = 48)** |
| --- | --- |
| **Exposure-adjusted overall incidence, n (IR per 100 PTY)** | **All grades** |
| Gastrointestinal toxicity | 24 (31.5) |
| Hypersensitivity | 16 (15.8) |
| Pancreatic toxicity (including isolated pancreatic enzyme elevations) | 15 (15.3) |
| Pancreatitis (clinical events) | 1 (0.7) |
| Hepatotoxicity (including laboratory terms) | 15 (13.2) |
| Myelosuppression | 14 (11.3) |
| Erythropenia (anemia) | 5 (3.6) |
| Neutropenia | 8 (6.0) |
| Thrombocytopenia | 10 (7.8) |
| Cytopenias affecting > 1 lineage | 1 (0.7) |
| Hemorrhage | 10 (8.4) |
| Edema and fluid retention | 9 (7.3) |
| Ischemic heart and CNS conditions | 7 (5.1) |
| Ischemic heart disease | 5 (3.6) |
| Ischemic CNS vascular conditions | 4 (2.8) |
| Cardiac failure (clinical events) | 1 (0.7) |
| Phototoxicity | 1 (0.7) |
| QTc prolongation | 1 (0.7) |

CNS, central nervous system; IR, incidence rate; PTY, patient treatment years; QTc, corrected QT internal.

**Table S7:** Arterial Occlusive Events by Preferred Term

|  | **All patients (n = 48)** | |
| --- | --- | --- |
| **Patients, n (%)** | **All grades** | **Grade ≥ 3** |
| Arterial occlusive events | 6 (12.5) | 3 (6.3) |
| Cerebrovascular accident | 3 (6.3) | 2 (4.2) |
| Carotid artery disease | 1 (2.1) | 0 |
| Coronary artery disease | 1 (2.1) | 1 (2.1) |
| Peripheral arterial occlusive disease | 2 (4.2) | 1 (2.1) |
| Peripheral artery occlusion | 1 (2.1) | 0 |
| Acute coronary syndrome | 1 (2.1) | 0 |
| Myocardial infarction | 1 (2.1) | 1 (2.1) |
| Ischemic stroke | 1 (2.1) | 1 (2.1) |
| Exposure-adjusted overall incidence, n (IR per 100 PTY) | 6 (4.4) | NA |

Adapted from Cortes JE, et al. *Leukemia*. 2024;38(7):1522-1533.(1) Creative Commons Attribution 4.0 International License (<https://creativecommons.org/licenses/by/4.0/>).

Reused with permission from the American Society of Hematology. © 2024 The Authors. All rights reserved. Officially licensed by ASH for distribution via Novartis Pharmaceuticals.

IR, incidence rate; NA, not available; PTY, patient treatment years.

**Table S8:** Patients with Arterial Occlusive Events

| **Age, years** | **Sex** | **Narrative** | **Baseline CV risk factors and relevant medical history** | **Prior TKIs** |
| --- | --- | --- | --- | --- |
| 68 | Male | Cerebrovascular accident (grade 3; day 917) 13 days after hospitalization due to COVID-19 and died 23 days post hospitalization due to pneumonia with COVID-19 as a contributing factor | **Past:** thrombocytosis  **Active:** Von Willebrand disease,  hypertension, degenerative aortic and mitral valve disease, hypercholesterolemia, hepatitis B, bradycardia, obesity | Imatinib,  dasatinib, and  nilotinib |
| 52 | Male | Patient discontinued asciminib (day 138) due to disease progression  Cerebrovascular accident (grade 1) occurred 2 days after patient discontinued asciminib | **Active:** diabetes, depression, insomnia, hypertension | Imatinib,  dasatinib,  nilotinib, and ponatinib |
| 77 | Male | Carotid artery disease (grade 1) occurred on day 590; no action taken with asciminib  The patient completed treatment with asciminib and continued to receive asciminib in posttrial access (day 1660 [54.5 months]) | **Active:** hypothyroidism, anemia,  hypertension, coronary artery disease | Imatinib, nilotinib, and  ponatinib |
| 76 | Male | Peripheral artery occlusion  Peripheral arterial occlusive  disease (grade 2) occurred on days 505 (grade 2; left side) 511 (grade 3; left side) and 602 (grade 3; right side); no action taken with asciminib    Coronary artery disease (grade 3) occurred on day 1274; no action taken with asciminib  The patient completed treatment with asciminib and continued to receive asciminib in posttrial access (day 2184 [71.8 months]) | **Past:** cerebrovascular  accident, sleep apnea syndrome  **Active:** hypertension | Imatinib,  dasatinib,  bosutinib, and  ponatinib |
| 64 | Male | Acute coronary syndrome (grade 2) occurred on day 1237 (40.6 months); no action taken with asciminib  Patient discontinued the trial on day 1774 (58.3 months) due to physician decision and continued to receive asciminib in posttrial access | **Past:** malaria, tobacco usage, and carbon monoxide poisoning  **Active:** lumbar spinal stenosis, spinal osteoarthritis, intervertebral disc protrusion, arteriosclerosis, renal artery stenosis, dry skin, benign prostatic hyperplasia, hypertension, and psoriasis | Ponatinib and nilotinib |
| 73 | Female | Peripheral arterial occlusive disease (grade 2) occurred on study day 625 (20.5 months)  Ischemic stroke occurred on day 980 (32.2 months; grade 2) and day 988 (32.5 months; grade 3)  The patient completed the trial and continued to receive asciminib in posttrial access (day 1093 [35.9 months])  Cerebrovascular accident (grade 4) and myocardial infarction (grade 4) occurred on day 1116 (36.7 months) and resulted in death | **Past:** skin neoplasm excision, hysterectomy, acute coronary syndrome, dyslipidemia, stent placement, ischemic heart disease prophylaxis, coronary angioplasty, myocardial infarction, and tendon disorder  **Active:** hypertension, hyperglycemia, overweight, myocardial necrosis, tachycardia | Imatinib, nilotinib, ponatinib, and bosutinib |

Adapted from Cortes JE, et al. *Leukemia*. 2024;38(7):1522-1533.(1) Creative Commons Attribution 4.0 International License (<https://creativecommons.org/licenses/by/4.0/>).

CV, cardiovascular; TKI, tyrosine kinase inhibitor.

**Table S9:** Summary of Pharmacokinetic Parameters

| **Treatment** | **Statistics** | **AUClast (hr*ng/mL)** | **AUCtau (hr*ng/mL)** | **Cmax (ng/mL)** | **Ctrough (ng/mL)** | **Tmax**  **(hr)** | **T1/2**  **(hr)** | **CL/F (L/hr)** | **Vz/F**  **(L)** | **Racc** |
| --- | --- | --- | --- | --- | --- | --- | --- | --- | --- | --- |
| **Cycle 1 day 1 (following first dose)** | | | | | | | | | | |
| **Asciminib 200 mg BID (n = 48)** | **n** | **48** | **39** | **48** | NA | **48** | **2** | **39** | **2** | NA |
|  | Mean (SD) | 15690.5 (5346.47) | 20654.7 (6228.80) | 3637.08 (1177.10) | NA | NA | NA | 10.54 (3.13) | 41.82 (3.43) | NA |
|  | CV% mean | 34.07 | 30.16 | 32.36 | NA | NA | NA | 29.71 | 8.20 | NA |
|  | Geo-mean | 14835.9 | 19797.4 | 3450.79 | NA | NA | NA | 10.10 | 41.75 | NA |
|  | CV% geo-mean | 34.96 | 30.10 | 34.27 | NA | NA | NA | 30.10 | 8.22 | NA |
|  | Median | 14760.0 | 20330.1 | 3365.00 | NA | 2.05 | 2.56 | 9.84 | 41.82 | NA |
|  | [Min; Max] | [7595.05; 28751.9] | [10024.3; 40705.8] | [1390.00; 6840.00] | NA | [0.95; 7.28] | [2.00; 3.12] | [4.91; 19.95] | [39.40; 44.24] | NA |
| **Cycle 2 day 1 (steady state)** | | | | | | | | | | |
| **Asciminib 200 mg BID (n = 48)** | **n** | **43** | **28** | **43** | **44** | **43** | NA | **28** | NA | **24** |
|  | Mean (SD) | 32497.6 (14937.2) | 41405.1 (20184.6) | 5939.3 (2559.45) | 3255.16 (1999.86) | NA | NA | 5.79  (2.58) | NA | 2.16 (0.61) |
|  | CV% mean | 45.96 | 48.75 | 43.09 | 61.44 | NA | NA | 44.58 | NA | 27.98 |
|  | Geo-mean | 29753.9 | 37733.6 | 5479.64 | 2799.71 | NA | NA | 5.30 | NA | 2.09 |
|  | CV% geo-mean | 43.65 | 45.12 | 41.83 | 59.86 | NA | NA | 45.12 | NA | 27.72 |
|  | Median | 28110.0 | 37092.1 | 4820.00 | 2685.00 | 2.00 | NA | 5.42 | NA | 2.11 |
|  | [Min; Max] | [10144.6; 84207.8] | [13473.3; 119753] | [2000.00; 15200.0] | [527.00; 11200.0] | [0.90; 7.03] | NA | [1.67; 14.84] | NA | [1.09; 3.80] |

AUC, area under the curve; BID, twice daily; CL/F, oral clearance; Cmax, maximum concentration; Ctrough, trough concentration; CV%, coefficient of variation; geo-mean, geographic mean; max, maximum; min, minimum; NA, not available; Racc, accumulation ratio;T1/2, half-life; Tmax, time to maximum concentration; Vz/F, apparent volume of distribution.

**Figure S1:** Study Design

Adapted from Cortes JE, et al. *Leukemia*. 2025;39(5):1124-1134.(2) Copyright © 2025, The Author(s).

Adapted from Hochhaus A, et al. *Leukemia*. 2025;39(5):1114-1123.(3) Creative Commons Attribution 4.0 International License (<https://creativecommons.org/licenses/by/4.0/>).

ALL, acute lymphoblastic leukemia; AP, accelerated phase; BID, twice daily; BP, blast phase; CML, chronic myeloid leukemia; CP, chronic phase; ECOG, Eastern Cooperative Oncology Group; MTD, maximum tolerated dose; Ph, Philadelphia chromosome; QD, once daily; RDE, recommended dose for expansion; TKI, tyrosine kinase inhibitor.

^a^ Patients without a T315I mutation and with relapsed, refractory, or intolerant disease were eligible after ≥ 2 prior TKIs. Patients with CML-BP or Ph+ ALL who had a cytopathologically confirmed diagnosis and were relapsed or refractory to ≥ 1 prior TKI or intolerant of TKIs were eligible.

^b^ Recommended dose for expansion has not been determined and no dose-expansion cohort was opened, but 5 additional patients with CML-BP/Ph+ ALL were enrolled in an enrichment cohort at 200 mg BID.

^c^ Dose expansion of asciminib + imatinib is being assessed in a separate phase 2 study (NCT03578367).

**Figure S2:** Consort Diagram

Adapted from Hochhaus A, et al. *Leukemia*. 2025;39(5):1114-1123.(3) Creative Commons Attribution 4.0 International License (<https://creativecommons.org/licenses/by/4.0/>).

ALL, acute lymphoblastic leukemia; AE, adverse event; AP, accelerated phase; BP, blast phase; CML, chronic myeloid leukemia; CP, chronic phase; Ph, Philadelphia chromosome.

^a^ Treatment ongoing as of the cutoff (March 14, 2023) in posttrial access, including patients who continued to receive asciminib in a rollover study or commercial availability.

^b^ Three patients were excluded due to having atypical or unknown *BCR::ABL1* transcripts at screening.

**Figure S3:** Overall Molecular Response Rate in All Molecular Response–Evaluable Patients^a^ Who Received Asciminib 150 or 160 mg BID,^a^ Including Those With Response at Baseline

Reused with permission from the American Society of Hematology. © 2024 The Authors. All rights reserved. Officially licensed by ASH for distribution via Novartis Pharmaceuticals.

BID, twice daily; IS, International Scale; MMR, major molecular response; MR^4^, *BCR::ABL1*^IS^ ≤0.01%; MR^4.5^, *BCR::ABL1*^IS^ ≤0.0032%.

^a^ In this trial, 5 and 6 patients received asciminib 150 and 160 mg BID, respectively. 1 patient in each cohort was excluded from efficacy analyses due to atypical/e1a2/e1a3/unknown *BCR::ABL1* transcripts.

^b^ Of evaluable patients who received asciminib 150 mg BID, 1 patient had MMR at baseline. All were ponatinib naive.

^c^ Of evaluable patients who received asciminib 160 mg BID, 1 patient had *BCR::ABL1*^IS^ ≤ 1% at baseline. 4 of 5 patients were ponatinib pretreated.

^d^ n = number of patients with a response; M = number of evaluable patients.

**Figure S4:** Incidence of Adverse Events Over Time^a^

Adapted from Cortes JE, et al. Leukemia. 2024;38(7):1522-1533.(1) Creative Commons Attribution 4.0 International License (<https://creativecommons.org/licenses/by/4.0/>).

LRTI, lower respiratory tract infection; URTI, upper respiratory tract infection.

^a^ Proportion of patients with on-treatment adverse events occurring during year 1, year 2, year 3, year 4, and year ≥ 5. Denominators are the number of patients on treatment at the beginning of the indicated time interval. A patient with multiple occurrences of an adverse event with the same preferred term in the same time interval is counted only once in that time interval.

**References**

1.	Cortes JE, Sasaki K, Kim DW, Hughes TP, Etienne G, Mauro MJ, et al. Asciminib monotherapy in patients with chronic-phase chronic myeloid leukemia with the T315I mutation after ≥1 prior tyrosine kinase inhibitor: 2-year follow-up results. Leukemia. 2024; 38:1522–33.

2.	Cortes JE, Lang F, Rea D, Hochhaus A, Breccia M, Goh YT, et al. Asciminib in combination with imatinib, nilotinib, or dasatinib in patients with chronic myeloid leukemia in chronic or accelerated phase: phase 1 study final results. Leukemia. 2025; 39:1124–1134.

3.	Hochhaus A, Kim DW, Cortes JE, Sasaki K, Mauro MJ, Hughes TP, et al. Asciminib monotherapy in patients with chronic myeloid leukemia in chronic phase without BCR::ABL1^T315I^ treated with at least 2 prior TKIs: phase 1 final results. Leukemia. 2025; 39:1114–1123.
